# Uric acid metabolism modulates diet-dependent responses to intraspecific competition in *Drosophila* larvae

**DOI:** 10.1016/j.isci.2022.105598

**Published:** 2022-11-15

**Authors:** Juliano Morimoto

**Affiliations:** 1 School of Biological Sciences, University of Aberdeen, Zoology Building, Tillydrone Avenue, Aberdeen AB24 2TZ, UK; 2 Programa de Pós-graduação em Ecologia e Conservação, Universidade Federal do Paraná, Curitiba 82590-300, Brazil

**Keywords:** Ecology, Biological sciences, Developmental biology

## Abstract

Intraspecific competition drives ecological specialization, and niche expansion. In holometabolous insects, larval intraspecific competition (“crowding”) has lasting fitness consequences for individuals and shapes adaptive responses. However, our understanding of the molecular profile of larval crowding responses remains allusive. Here, I used a genetic construct in *Drosophila melanogaster* with disrupted uric acid metabolism (*uro*^−^) to demonstrate the role of the uric acid metabolism—and its interactive effects with larval crowding, diet, and urea concentration—on female oviposition and larval development. *Uro*^−^ larvae developed faster in sugar-rich diets. However, *uro*^−^ larvae pupated at lower heights in protein- and sugar-rich diets in crowded larval conditions and showed slower development in protein-rich mixed-genotype environments. *uro*^−^ did not affect female oviposition nor larval pupation success. Overall, this study provides the first step toward an integrated understanding of the molecular pathways underpinning the responses to intraspecific competition in holometabolous insects.

## Highlights

• Uric acid metabolism (UAM) is modulated by larval crowding and diet in insects
• UAM disruption led to the physiological accumulation of uric acid in *Drosophila* adults
• UAM disruption had no effect on female oviposition
• UAM disruption led to diet-dependent responses to larval crowding

## Graphical abstract

**Figure:**
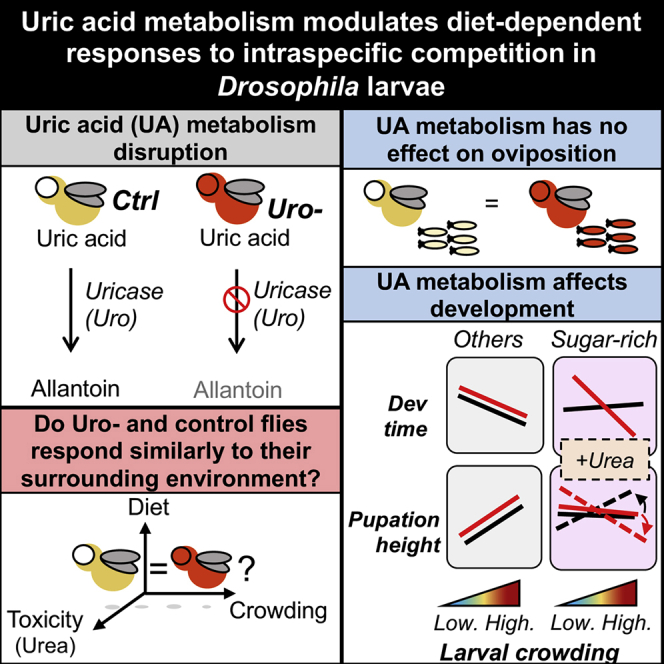

## Introduction

Conspecifics within a population can compete for access to limited resources which in turn limits the ability of individuals to survive and reproduce (“intraspecific competition”). As a result, intraspecific competition creates a selective pressure that leads to a range of adaptations across biological levels of organization, from physiological responses to stress (molecular) to cannibalism (behavioral).^1^^,^^2^ Ultimately, intraspecific competition (and the selective pressure created by it) can lead to adaptation to novel resources and niche expansion.^3^^,^^4^^,^^5^^,^^6^ Studies in insects and fish have shown that populations experiencing high intraspecific competition can adapt to unfavorable environments more rapidly than populations under low intraspecific competition, highlighting the importance of intraspecific competition for adaptation.^7^^,^^8^ Therefore, intraspecific competition remains a central concept in animal ecology.^9^^,^^10^^,^^11^^,^^12^^,^^13^

In holometabolous insects, the intraspecific competition during early development affects the quantity and quality of resources that individuals can acquire to invest in life-history traits in adulthood.^14^^,^^15^ In fact, intraspecific competition at the larval stage of insects (“larval crowding”) is an essential but often overlooked source of individual phenotypic variation and adaptive potential.^7^^,^^16^^,^^17^^,^^18^^,^^19^ Studies in *Drosophila melanogaster* have been invaluable in uncovering the plastic responses to larval crowding. For instance, larvae developing in crowded conditions have upregulated expression of heat-shock proteins in response to the stress of the developmental environment,^20^^,^^21^^,^^22^ which may be involved in a hormesis-like response that leads to increased lifespan and fitness benefits in broader ecological contexts.^21^^,^^23^^,^^24^^,^^25^

Two factors are likely to be the primary sources of stress for larvae in crowded environments: dietary protein scarcity and toxic waste accumulation. Protein is an essential nutrient for the growth of the developing larvae and diets which are protein limited lead to significant developmental delays.^26^^,^^27^^,^^28^^,^^29^^,^^30^ Larval crowding leads to protein limitation, therefore acting as a dietary stressor to the developing larvae.^23^ Similarly, when individuals feed in a confined environment, toxic waste compounds accumulate and can further decrease the quality of the diet above and beyond the depletion of protein. Larvae excrete toxic compounds such as urea which accumulates in the diet, particularly when larval crowding is high, leading to toxic effects to the developing individuals and imposing an additional layer of stress.^20^^,^^31^^,^^32^^,^^33^^,^^34^ These two sources of stress—i.e., diet and toxic waste—interact and impose a strong selective pressure. Studies have shown that larvae evolving under high-crowding larval environments have an increased ability to convert nutrients into growth and higher tolerance to urea.^32^^,^^34^^,^^35^^,^^36^^,^^37^^,^^38^ Thus, it is reasonable to expect that an adaptive response to larval crowding requires the integration of complex environmental information related to the crowding levels, diet, and toxicity of the developmental environment.

The uric acid pathway can integrate the inputs from both nutrition and toxic waste compounds, becoming an important molecular candidate for the responses observed in high larval crowding. This is because the uric acid pathway can respond to protein intake *and* regulate the production of toxic waste production such as excreted urea.^39^^,^^40^ In fact, the expression of the enzyme *uricase* (*uro*), a key enzyme in the uric acid pathway which converts uric acid into allantoin, was differentially increased for larvae developing in crowded environments,^20^^,^^41^ corroborating the potential role of *uro* in the responses to larval crowding. Moreover, in *D. melanogaster*, *uro* knockdown leads to the accumulation of uric acid and concretions in the Malpighian tubules, particularly in protein-rich diets.^40^ *Uro* knockdown phenotypes were ameliorated upon the downregulation of the insulin-like signaling, highlighting the diet-dependent regulation of uric acid metabolism.^40^ To date, however, studies have focused on the role of *uro* in relatively simple environments which do not necessarily reflect the complexity and dynamic context in which larval compete and develop. For example, Henry et al., (2018) showed that *uro* is upregulated in larvae from high larval crowding, but did not explicitly manipulate the diet or the toxicity levels of the developmental environment. Likewise, Lang et al., (2019) manipulated diet and *uro* activity levels but maintained larval crowding levels constant. Thus, a more complex but also more realistic experimental design, which focuses on the interactions between larval crowding, diet, and toxicity levels is needed for an ecologically relevant understanding of the role of *uro* in the responses to larval crowding.

**Figure 1:**
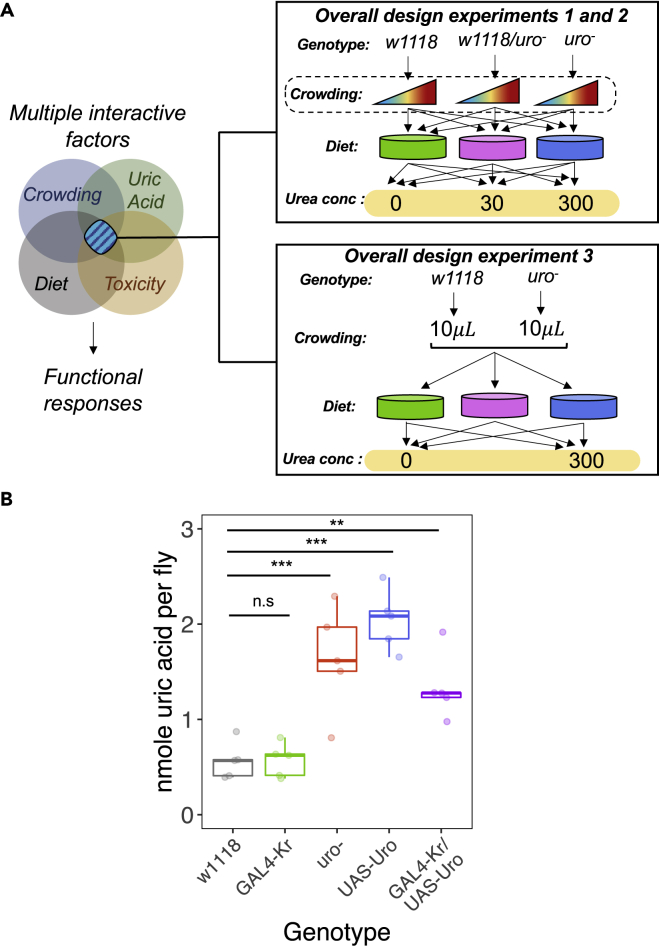
Uric acid metabolism role in larval responses (A) Overall experimental design. *Experiments 1* and *2* investigated female oviposition and larval development, respectively. Note that, for *Experiment 1*, there was no larval crowding manipulation (dashed boxed) as females were allowed to oviposit freely; the larval crowding manipulation only applies to *Experiment 2* (see also Figure S1). (B) Uric acid concentration across fly genotypes. There was a statistically significant difference in the accumulation of uric acid (ANOVA: F_4,20_ = 17.837, p < 0.001) whereby controls w1118 and GAL4-*Kr* had substantially lower uric acid concentrations than the homozygotes *uro*^*-*^*,* UAS-Uro and the GAL4-*Kr* x UAS-Uro cross. See also Tables S1 and S3.

Here, I addressed this gap by investigating the role of uric acid metabolism in the developmental responses of *D. melanogaster* larvae experiencing varying larval crowding and across diets varying in both protein content and urea concentration levels. To ensure the study is ecological relevant, I manipulated larval crowding levels using a pioneering protocol that generates a continuous distribution of the variance in larval crowding [as opposed to the traditional simple dichotomous treatments of “high” vs “low” crowding as in e.g.,^42^^,^^43^^,^^44^ (Figure 1A). This approach allowed me to represent larval crowding as a continuous variable as opposed to a discrete one. More importantly, the manipulation of larval crowding done here was determined based on the number of eggs females laid in one of the experiments (see details above) as well as the larval crowding levels observed in a natural *D. melanogaster* population.^45^ This ensured that the findings can be interpreted within the ecological context of the species. Using a genetic construct with impaired *uricase* (*uro*) activity, I manipulated the yeast-to-sugar ratio (Y:S ratio) of the larval diet, the concentration of urea (0, 30, 300 mM), and the genotype the larvae and measured female egg-laying behavior, larval developmental time, pupation height and pupation success (i.e., the number of pupae relative to the number of eggs seeded) as a proxy of fitness (see Table S1). I also measured the ability of larvae to develop in mixed-genotype environments across diets and urea concentration levels and measured the proportion of fast-emerging individuals of each genotype as a proxy of developmental speed. In *Drosophila,* developmental time is known to correlate with reproductive fitness^46^^,^^47^ while pupation height is modulated by the quality of the larval developmental environment—including larval competition and diet—and also correlates with fitness.^48^^,^^49^^,^^50^^,^^51^ Under the assumption that *uro* affected the physiological state of individuals, I predicted that females with impaired *uro* would lay fewer eggs, particularly in protein-rich and/or urea-rich diets. Moreover, I predicted that larvae with impaired *uro* would display longer developmental times, higher pupation height, and lower pupation success relative to controls, mimicking the behavior of larvae and adults from crowded environments. The magnitude of these effects would be more evident in larvae with impaired *uro* developing in protein-rich diets due to the known effects of *uro* knockdown (see above).^40^ The findings presented here provide the first direct evidence for the role of the uric acid metabolism on the responses to larval crowding, shedding light onto the role of purine metabolism on the development of holometabolous insects.

## Results

### *Uro* impairment leads to uric acid accumulation

In the absence of functional *uricase*, its substrate (i.e., uric acid) accumulates. I first tested whether the *D. melanogaster* genetic construct *PBac{WH}Uro*^*04888*^ (henceforth *uro*^*-*^) accumulated uric acid as evidence that the construct was suitable for the subsequent investigations of the role of uric acid metabolism on larval crowding responses. I measured the average uric acid concentration (nmole/fly) of control *w1118*, homozygotes *uro*^*-*^ and the GAL4-UAS lines for RNAi (i.e., *Krüppel*-GAL4 and UAS-Uro RNAi; see STAR methods for details). I expected that *Krüppel*-GAL4 and UAS-Uro RNAi controls to have low uric acid accumulation while the cross *Krüppel*-GAL4 x UAS-Uro RNAi to have similar uric acid accumulation levels of *uro*^*-*^ flies. Flies of all genotypes were reared in the balanced diet (see Table S1 for recipe, Figure 1A) from egg to adult upon which the uric acid quantification was performed. As expected, both *uro*^*-*^ and the RNAi cross-accumulated uric acid relative to w1118 controls (Figure 1B). There was also an unexpected accumulation of uric acid in the UAS-Uro RNAi control likely due to “leakage” in the regulation of the UAS promoter that leads to expression of the RNAi in the absence of GAL4 (see Discussion for details). Overall, the results demonstrate that *uro*^*-*^ accumulates uric acid as expected should *uricase* be functionally absent, as opposed to controls w1118 and the GAL4-*Kr* which had functional *uricase* and displayed low uric acid levels.

### Diet composition and urea concentration modulate oviposition behavior independent of uric acid metabolism

**Figure 2:**
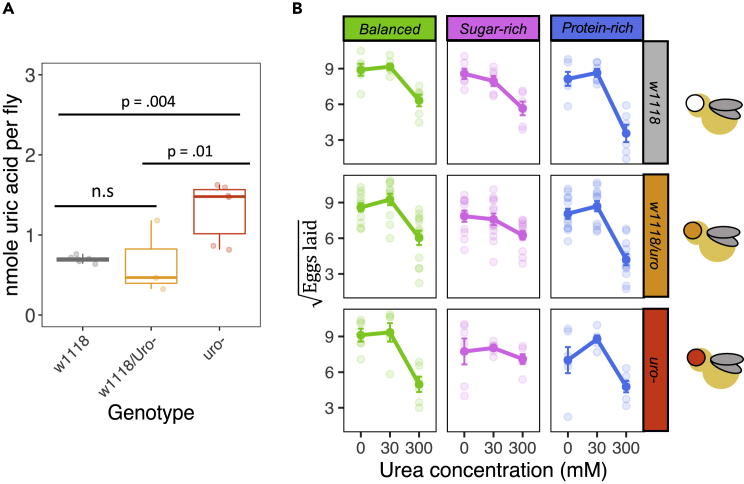
Uric acid metabolism is disrupted but does not affect female oviposition behavior (A) Uric acid concentration across fly genotypes. There was a statistically significant difference in the accumulation of uric acid (ANOVA: F_2,12_ = 7.621, p = 0.007) whereby controls w1118 and w1118/*uro*^*-*^ had substantially lower uric acid concentrations than the homozygotes *uro*^*-*^. (B) Female oviposition across the genotypes was unaffected by uric acid metabolism disruption but responded to the toxicity levels of the substrate. See also Figure S1 and Table S3.

Next, I investigated how *uro* could affect female oviposition. In insects, female oviposition is a way in which females can modulate the larval crowding experienced by their offspring.^52^ If females were in a physiological state of uric acid accumulation, it is possible that these females could decrease egg laying to minimize larval crowding effects to their progeny. To test this, five mated females were placed in vials containing one of the three diets (balanced, protein- and sugar-rich) and one of the three urea concentration levels (0, 30, 300 mM) in a fully factorial design (see Table S1 for the recipe, Figure 1). Females were either of control (w1118), heterozygotes (w1118*/uro*^*-*^), and homozygote *uro*^−^ genotypes. Uric acid accumulated in *uro*^*-*^ compared with either controls or heterozygotes as expected (Figure 2A). Despite this, female genotype had no statistical effect on oviposition (F_2, 199_ = 1.00, p = 0.390; Table S2). There was a statistically significant interaction between diet and the non-linear (quadratic) term of urea concentration (*Diet∗[Urea]*^*2*^: F_2, 199_ = 3.559, p < 0.001). This effect emerged because females of all genotypes laid a similar number of eggs when diets contained 0 mM or 30 mM or urea, but laid substantially fewer eggs in all diets which contained 300mM of urea (Tables S2 and S3). However, this decline in oviposition based on urea concentration was sharper in the balanced and protein-rich diets, while the decline in oviposition in sugar-rich diets was almost linear with increasing urea (Figure 2B). There were also statistically significant main effects of diet (*Diet*: F_2, 199_ = 8.667, p < 0.001) and the non-linear term of urea concentration (*[Urea]*^*2*^: F_2, 199_ = 9.800, p < 0.001; Table S2). Overall, these results show that uric acid metabolism does not influence female oviposition behavior but that females modulate their oviposition based on cues from the environment such as nutrient content and toxicity of the substrate.

### Uric acid metabolism modulates larval development and pupation height as a response to intraspecific competition in a diet-specific fashion

**Figure 3:**
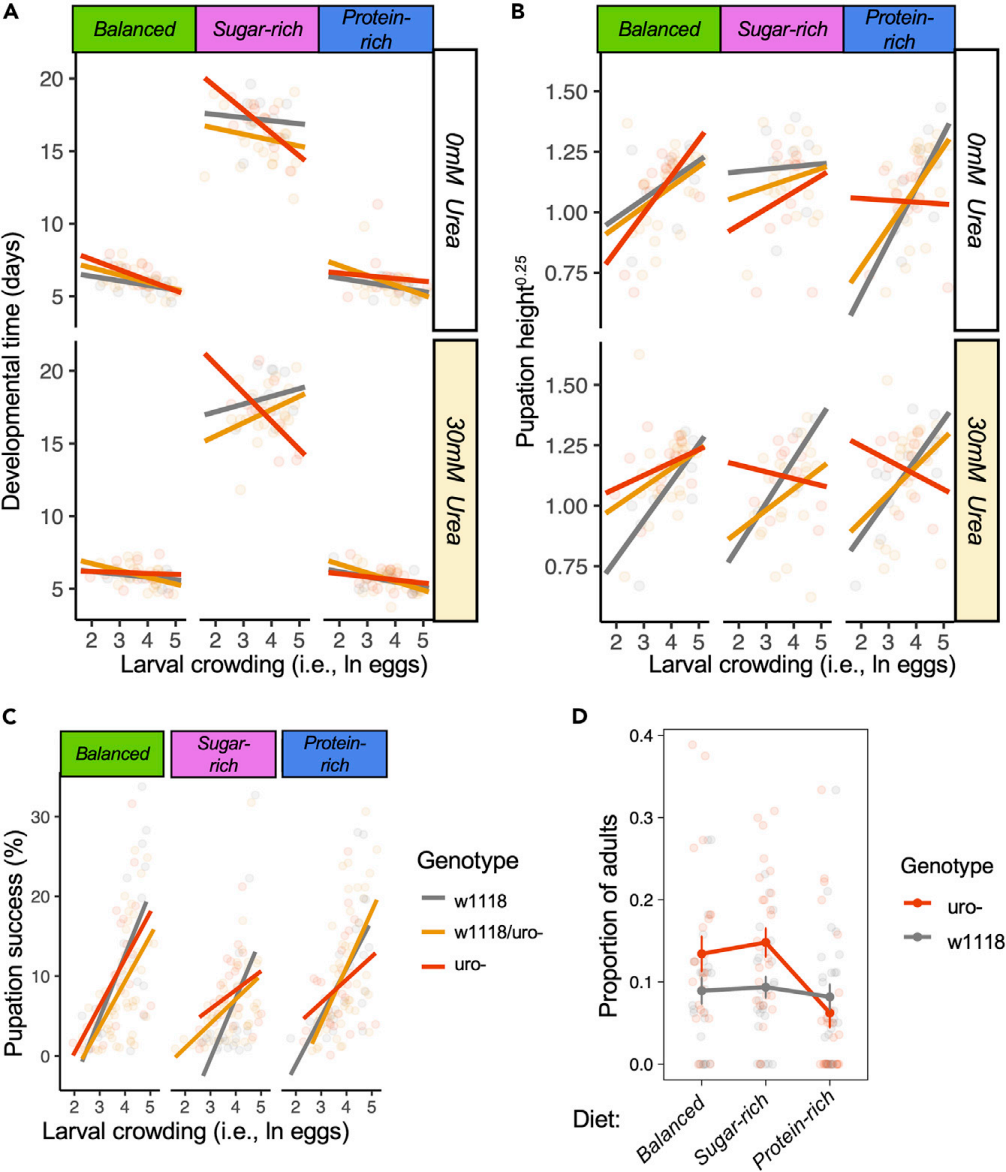
Uric acid metabolism effects on larval development (A) The relationship between larval crowding and larval developmental time across diets and urea concentration (0 and 30mM). (B) The relationship between larval crowding and pupation height across diets and 0 and 30mM urea concentration. Pupation height was transformed (power of 0.25; see “STAR methods”). (C) The relationship between larval crowding and pupation success (as the percentage of pupae) across diets. (D) Developmental speed in mixed-genotype developmental environments with varying diets and 0mM of urea. This proxy was measured as the proportion of adults of a given genotype that were first to emerge as adults (i.e., within a 4-h window). Note that for panels a-c, larval crowding was natural log (i.e., ln-transformed) to improve model fit (see “STAR methods” for details). Treatments containing 300 mM of urea induced high mortality (>70%, see “STAR methods”) and were removed from the analysis. See also Table S4.

Next, I investigated the role of uric acid metabolism on larval developmental traits such as larval development, pupation height, and pupation success. The data showed a statistically significant three-way interaction between parental genotype, larval crowding, and diet on developmental time (*Genotype∗ Crowding ∗Diet:* F_4, 270_ = 5.086, p = 0.001). This effect emerged because the slope of the relationship between larval crowding and developmental time was close to zero (i.e., flat) for all parental genotypes in protein-rich and balanced diets. However, for the sugar-rich diet, the slope of the relationship between larval crowding and developmental time was flat for both control w1118 and heterozygotes w1118*/uro*^*-*^ but negative for *uro*^*-*^ flies (Figure 3A). The negative slope between larval crowding and developmental time was more accentuated (i.e., more negative) in 30mM compared with 0mM of urea concentration for *uro*^*-*^ flies (*[Urea]∗Diet:* F_2, 270.8_ = 7.179, p < 0.001) (Figure 3A). There were also statistically significant two-way interactions between parental genotype and diet (*Genotype∗Diet:* F_4, 269.7_ = 3.263, p = 0.012) which was largely driven by faster developmental time for w1118 flies in protein-rich and balanced diet but longer developmental time for w1118 in the sugar-rich diet relative to heterozygotes w1118*/uro*^*-*^ and *uro*^*-*^ flies. There also were statistically significant main effect of larval crowding (*Crowding:* F_1, 268.1_ = 26.792, p < 0.001), and diet (*Diet:* F_2, 270.3_ = 3269.84, p < 0.001) on developmental time (Table S4).

The data also showed a statistically significant three-way interaction between parental genotype, larval crowding, and diet on pupation height (*Genotype∗ Crowding ∗Diet:* F_4, 241.3_ = 2.413, p = 0.049; Table S4) and the two-way interaction between diet and urea concentration on pupation height (*[Urea]∗Diet:* F_4, 243.1_ = 3.764, p = 0.025). Together, these results were driven by the fact that pupation height increased with larval crowding across all diets for both control w1118 and heterozygotes w1118*/uro*^*-*^ flies, as well as for the *uro*^*-*^ flies in balanced diet. However, this relationship was overall flat or negative for *uro*^*-*^ flies in sugar-rich or protein-rich diets, particularly in diets with 30 mM of urea (Figure 3B). There also was an interaction between genotype and larval crowding on pupation success (*Genotype∗Crowding:* F_2, 269.3_ = 5.105, p = 0.007) which was driven by an overall lower pupation success with increasing larval crowding in *uro*^*-*^ flies relative to w1118 and w1118*/uro*^*-*^ (Figure 3C). There was also a statistically significant main effects of larval crowding (*Crowding:* F_1, 270_ = 207.181, p < 0.001) and diet (*Diet:* F_2, 269.4_ = 13.379, p < 0.001) on pupation success (i.e., the number of pupae relative to the number of eggs seeded). No other main or interactive effects of genotype were statistically significant. Overall, the findings show that uric acid metabolism modulates the magnitude of the effects of larval crowding on larval development in a diet-dependent fashion.

### Uric acid metabolism modulates developmental speed in mixed-genotype environments

The data above showed that the uric acid metabolism is involved in the regulation of larval responses to the conditions of the developmental environment. In nature, it is likely that multiple genotypes interact in the developmental environment, creating the potential for positive and negative interactions between genotypes that modulate larval development. To gain insights into how uric acid metabolism can play a role in the development of flies in mixed genotype environments, I created mixed genotype environments by seeding the same volume of eggs with synchronized development time from both control w1118 and *uro*^*-*^ genotypes in balanced, sugar-rich, and protein diets containing either 0 mM or 300 mM of urea. I then measured the proportion of adults of each genotype that were the first to emerge (“fast-emerging adults”) as a proxy of developmental speed. Naturally, fast-emerging adults may have fitness advantages in a population by e.g., gaining access to better patches or mates [although this is not the case for artificially selected lines for fast development which suffer fitness costs; see e.g.,^47^^,^^53^]. The data showed that *uro*^*-*^ had faster development in mixed genotype environments (i.e., higher proportion of fast-emerging adults) with one exception: *uro*^*-*^ developmental speed was significantly slower in protein-rich diets, which led to the proportion of fast-emerging adults to be similar between w1118 and *uro*^*-*^ genotypes in protein0rich diets (*Genotype∗Diet*: F_2, 146_ = 3.843, p = 0.023, Figure 3D). There was also a significant main effect of diet (*Diet*: F_2, 149_ = 7.739, p < 0.001) and genotype (*Genotype*: F_1, 148_ = 4.616, p = 0.033) on the proportion of fast-emerging adults. All larvae of both genotypes in 300mM urea died (see STAR methods).

## Discussion

This study investigated the role of uric acid metabolism and in particular, *uricase,* in the responses of *D. melanogaster* larvae to varying levels of larval intraspecific competition and types of developmental environments. This is, to my knowledge, the first empirical study to directly measure the role of a metabolic pathway in the responses to larval intraspecific competition building upon previous studies in the field [e.g.,^41^^,^^54^]. I showed that the disruption of the uric acid metabolism, which led to uric acid accumulation, shortened developmental time, and decreased pupation height of larvae developing in sugar-rich and protein-rich diets. Moreover, the disruption of the uric acid metabolism also led to lower pupation success for larvae developing in sugar-rich diets. Lastly, uric acid metabolism was shown to be important for larval development in mixed-genotype environments in a diet-dependent fashion, whereby there was a significantly lower proportion of fast-emerging adults in protein-rich diets, but not in sugar-rich and balanced diets. Importantly, uric acid metabolism did not affect female oviposition behavior, which was primarily modulated by diet and the concentration of urea in the substrate. There was no evidence that uric acid metabolism interacted with urea concentration to modulate the responses to the larval developmental environment, suggesting that the uric acid metabolism primarily interacts with the nutrition and the intraspecific competition levels of the larval environment to shape larval plastic developmental responses.

The disruption of the uric acid metabolism and the subsequent uric acid accumulation shortened developmental time and decreased pupation height of larvae developing in sugar-rich and protein-rich diets. This effect was similar for *uro*^*-*^ larvae developing in sugar-rich 0mM or 30mM of urea for developmental time, and slightly stronger for *uro*^*-*^ larvae in sugar-rich and protein-rich diets with 30mM compared with 0mM urea for pupation height. These results suggest that the effects of uric acid metabolism disruption on life-history traits are likely a result of internal physiological effects of the accumulation of uric acid, although in some cases, the toxicity of the media due to the presence of low amounts of urea can accentuate the effects of internal uric acid accumulation. Future studies will help uncover the interplay between external sources of urea and internal accumulation of uric acid. Importantly, the data also suggest that the effects of uric acid on larval developmental time are more evident for larvae developing in resource-poor diets in higher larval crowding, which is in theory, the most adverse conditions for larval development. Indeed, sugar-rich diets are relatively resource-poor diets for the larvae of *Drosophila* and other flies [e.g.,^26^^,^^27^^,^^29^^,^^55^^,^^56^], unless the diet contains microbes that can act as a nutrient (e.g., amino acid) supplements.^57^^,^^58^^,^^59^ Furthermore, high larval crowding and/or high-sugar diets increase the oxidative stress of the larvae and decrease pupation height.^20^^,^^21^^,^^48^^,^^50^^,^^60^^,^^61^^,^^62^^,^^63^^,^^64^ Moreover, the disruption of the uric acid metabolism is known to induce oxidative stress via NAPH oxidase (NOX)^40^ creating additional physiological stress to the developing larvae. Thus, our data support a model in which the disruption of the uric acid metabolism can help larvae develop faster in highly competitive, resource-poor diets at a cost of higher oxidative stress (Figure 3B) (see later in discussion).

I showed that the disruption of the uric acid metabolism led to the accumulation of uric acid (Figures 1B and 2A). This finding is in line with previous literature which showed that *uro* knockdown leads to concretions in the hindgut and Malpighian tubules that in turn, leads to developmental delays and higher mortality, effects that are stronger in protein-rich diets.^40^ The data presented here show that pupation height (a sign of oxidative stress; see above) decreased as larval crowding increased in sugar-rich and protein-rich diets. Moreover, the increase in pupation success with larval crowding was less accentuated (i.e., shallower slopes) for the *uro*^*-*^ in sugar-rich and protein-rich diets. Therefore, it is possible that the accumulation of uric acid led to higher oxidative stress (which was compounded by increasing larval intraspecific competition) and resulted in relatively lower pupation height and pupation success imbalanced diets, but particularly so in protein-rich diets. Interestingly, I showed that, in a mixed-genotype environment, *uro*^*-*^ larvae had slower developmental speed relative to controls in protein-rich (but not balanced or sugar-rich) diet (Figure 3D). The concretions formed by the accumulation of uric acid may hamper the ability of larvae to convert nutrients and excrete toxic waste in protein-rich diets, resulting in relatively slower developmental speed compared with controls. These findings align with previous studies showing that larval competitive ability depends upon food utilization and nutrient conversion to biomass.^35^

In many insects, female oviposition is the one and only opportunity for parental care.^65^^,^^66^ Therefore, female oviposition is expected to be under selection, relying on a range of internal (physiological) and external (environmental) inputs for optimum response.^67^^,^^68^ In *Drosophila,* females are known to bias their oviposition to sugar-rich diets^69^ [but see also^70^] showing ability to integrate inputs that affect oviposition behavior. Furthermore, *Drosophila* females prefer to oviposit in substrates that had been previously oviposited or that were used by larvae.^71^^,^^72^ I predicted that females with accumulated uric acid could respond by avoiding laying eggs in substrates with high urea concentration (a potential sign of high larval concentration) and in high-protein diets as a result of their disbalanced physiological state. Moreover, I expected females of all genotypes to lay fewer eggs in diets with high urea concentration as this could signal a saturated developmental environment with high intraspecific competition and poor nutrition larval development. While the data does not support a role of uric acid metabolism in modulating female oviposition, the results confirmed previous findings of the literature in that females avoid ovipositing in diets containing high concentration of urea, likely due to the toxicity of the diet for the larvae.^33^ Surprisingly though, oviposition avoidance of toxic diets was less accentuated in the sugar-rich diet, which is the poorest substrate nutrient-wise for the developing larvae.^26^^,^^70^ In fact, compared with diets with 0 mM or 30 mM of urea, which still support larval development, females were relatively more likely to oviposit in a prohibitively toxic diet with 300 mM when the diet was sugar-rich as opposed to balanced or protein-rich. This is important because the experiment on larval development showed that the sugar-rich 300 mM diet is lethal to the larvae and therefore, if female responses were perfectly adaptive, females should avoid sugar-rich 300 mM as the oviposition substrate. Intermediate urea levels did not affect female oviposition suggesting that females may use other chemical cues to differentiate between fresh and previously used substrate for oviposition.^71^^,^^72^^,^^73^

It is important to mention the limitations of this study, which will serve as a guide for future investigations in the field. First, the measurements of developmental time used here relied on the pupation of the first larvae. As a result, the metric used here does not measure the impact of uric acid on changes in the spread (i.e., variance) of pupation, although it does capture the average delay in developmental time. It will be interesting for future studies to expand the range of metrics that are used to assess the effects of uric acid on larval development. Second, the homozygote UAS-Uro RNAi line used here accumulated uric acid in similar levels to that of the *uro*^*-*^ and GAL4-UAS crosses (Figure 2A). UAS RNAi constructs are known to be “leaky” and display a strong knockdown phenotype even in the absence of GAL4 expression.^74^^,^^75^^,^^76^ Therefore, it is likely that the UAS-Uro RNAi used here is leaky. Note however that the GAL4-UAS cross had an equivalent high accumulation of uric acid, as did the *uro*^*-*^ line. Thus, despite the leaky UAS RNAi line, the findings presented here derive from the impairment of uric acid metabolism.

In conclusion, I showed that the uric acid metabolic pathway is an important hub for the integration of various environmental signals that affect larval responses to diet and intraspecific competition levels. The purine metabolism—to which the uric acid metabolism belongs—is a cornerstone biochemical pathway for larval metamorphosis and growth^77^^,^^78^ as well as the relationship between insects and its symbionts.^79^ Thus, demonstrating the functional role of the uric acid metabolism on larval responses to conditions in the developmental environments provides an important step change toward an integrated eco-physiological understanding of insect development.

### Limitations of the study

This study focused primarily on responses at the developing stages of the *Drosophila* life cycle. Whether or not similar effects of uric acid metabolism disruption are present in adult traits related to fitness and population survival (e.g., lifespan and reproduction) remain the subject of further studies. Moreover, as mentioned in the discussion, this study does not establish whether the internal accumulation of uric acid is solely derived from uricase disruption or is compounded by the intake of urea from the diet. While this will not change the interpretation of the results, it will provide a nuanced understanding of the links between the toxicity of the environment and the (linear or non-linear) physiological accumulation of uric acid. Finally, the RNAi line used here likely has leakage (see Discussion) and therefore, better constructs (possibly using CRISPR-Cas9) will improve our ability to conduct targeted experiments to understand the role of uric acid metabolism on insect development.

## STAR★Methods

### Key resources table

**Table:** 

REAGENT or RESOURCE	SOURCE	IDENTIFIER
**Chemicals, peptides, and recombinant proteins**		

Agar	(Merck) Sigma-Aldrich®	Cat# 05040
Sucrose	(Merck) Sigma-Aldrich®	Cat# 1076515000
Brewer’s yeast	MP Biomedicals™	Cat# 0290331225
Urea	(Merck) Sigma-Aldrich®	Cat# U5378
Petri dishes	Gosselin™	Cat# 15468794
Organic grape juice concentrate (1L)	VinClasse®	N/A
Flystuff (59–101) Embryo Collection Cage For 100mm Petri Dishes	Scientific Laboratory Supplies	Cat# FLY1214
Flystuff (32-113RL) Drosophila Vials Reload Narrow (25 × 95cm) Polypropylene 5 Trays of 100 Vials	Scientific Laboratory Supplies	Cat# FLY1102

**Critical commercial assays**		

Uric acid kit	(Merck) Sigma-Aldrich®	Cat# MAK077

**Deposited data**		

Raw data used in this work	This study	https://doi.org/10.5061/dryad.b8gtht7gp

**Experimental models: Organisms/strains**		

*w1118*	BDSC	Cat# 6326
*PBac{WH}Uro*^*f4888*^	BDSC	Cat# 18814
*w[∗]; Df(2R)247/CyO, P{w[+mC]=GAL4-Kr.C}DC3, P{w[+mC]=UAS-GFP.S65T}DC7*	BDSC	Cat# 7155
*y[1] sc[∗] v[1] sev[21]; P{y[+t7.7] v[+t1.8]=TRiP.HMC06403}attP40*	BDSC	Cat# 67300

**Software and algorithms**		

R software	R Development Team	(R Core Team, 2019)
*lme4*	–	(Bates et al., 2007)
lmerTest	–	(Kuznetsova et al., 2017)
ggplot2	–	(Wickham, 2016)

### Resource availability

#### Lead contact

Further information should be directed to and will be fulfilled by the lead contact, Dr. Juliano Morimoto (juliano.morimoto@abdn.ac.uk).

#### Materials availability

This study did not generate new unique *Drosophila* genetic constructs.

### Experimental model and subject details

#### Fly stocks and genetic lines

I used four strains from the Bloomington Drosophila Stock Center (BDSC): control stock *w1118*, the *PBac{WH}Uro* ^*f4888*^ (henceforth *uro*^*-*^), w[∗]; Df(2R)247/CyO, P{w[+mC] = GAL4-Kr.C}DC3, P{w[+mC] = UAS-GFP.S65T}DC7 (henceforth ‘GAL4-*Kr*’) and y[1] sc[∗] v[1] sev[21]; P{y[+t7.7] v[+t1.8] = TRiP.HMC06403}attP40 (henceforth ‘UAS-Uro’). All strains were maintained in large population cages (>1,000 individuals) with *ad libitum* food. Strain stock center codes are listed in the key resources table.

### Method details

#### Stock maintenance and experimental conditions

Fly stocks were maintained, and all experiments hereby described were conducted, in a controlled temperature room at 23°C with 12:12 light:dark cycles and average humidity of 50%.

#### Uric acid assay

Fifty males and fifty females of each of the strains above were placed bottles with ca. 15mL of balanced diet and allowed to mate for 48h (N = 5 replicate groups per genetic line), after which adults were discarded and eggs allowed to develop to adulthood. Within 6 h of emergence, adults were collected, sexed, and maintained in single sex groups for 5 days. Five males and five females were randomly selected and placed in vials with ca 5 mL of balanced diet for 48h. The crosses were arranged as following: (1) w1118 x w1118 (N = 5), (2) www18 x *uro*^*-*^ (N = 3) (3) *uro*^*-*^ x *uro*^*-*^ (N = 5) (4) GAL4-*Kr* x GAL4-*Kr* (N = 5) (5) UAS-Uro x UAS-Uro (N = 5) and (6) GAL4-*Kr* x UAS-Uro (N = 5). Vials were reserved for 15 days until all adults had emerged. Ten adult females of each cross line were randomly selected for uric acid quantification. I homogenised the females in 110 *μ*L of 1M phosphate buffer (pH 7.4) and centrifuged at room temperature for 10 min at 15,000g. I removed the supernatant for uric acid quantification, which was conducted using the Uric acid kit (Sigma Aldrich). Six uL of fly homogenate were diluted with 44 *μ*L of uric acid assay buffer and mixed with 50uL of mix solution. For 50 *μ*L of mix solution, I mixed 2 *μ*L of uric acid assay probe, 2uL of uric acid assay enzyme, and 46 *μ*L of uric acid assay buffer as instructed by the manufacturer. 96-well plates were then incubated at 30°C for 30 min and absorbance was read at 570nm. Concentration of uric acid was compared against a standard curve generated alongside with the sample measurements and divided by 10 to obtain the concentration in nanomoles of uric acid per fly (nmole per fly). The same protocol was used to quantify uric acid after backcrossing in control w1118 (N = 6), heterozygote w1118/*uro*^*-*^ (N = 3) and *uro*^*-*^ lines (N = 6) (Figure 2A).

#### Oviposition and larval development experiments

Based on the results of the uric acid quantification, I used the *uro*^*-*^ genetic line to investigate the behavioral and developmental effects of uric acid metabolism. To do this, I firstly backcrossed *uro* into the w1118 population stock for eight generations to ensure genetic background homogeneity. Backcrossing had no effect on the differences in uric acid concentration between control w1118 and *uro*^*-*^ genetic lines (Figure 2A). The backcrossed lines were then maintained in large populations (>1,000 individuals) as mentioned previously, from which eggs were collected. I collected eggs from the stock populations using an oviposition device composed of a 90 mm Petri dish covered with 5 mL of a 1.5% gel-based solution containing agar (Sigma Aldrich) and commercial grape juice (VinClasse) A thin layer of yeast paste was spread onto the agar:juice solution after it was set to stimulate oviposition. The oviposition device was added to the large cages of both w1118 and *uro*^*-*^ strains for 8 h, after which the device was retrieved, and the eggs were brushed off of the agar using a soft brush and distilled water. The egg-water solution was deposited into a 15 mL Falcon tube, where the eggs were allowed to set at the bottom of the tube. The egg-water solution was then washed three times with distilled water, after which 10 *μ*L of eggs were ‘squirted’ into vials containing 5 mL of standard diet as described previously.^42^^,^^80^^,^^81^ Vials were stored in a controlled environment as described above until development was completed. When adults emerged, they were collected within 5 h to ensure that males and females were unmated. Adults were kept for five days in same-sex groups of 20–25 individuals in vials containing 5 mL of standard food and 2–3 yeast granules. I then placed 60 males and 60 females in an egg collection cage that was covered with the oviposition device as described above. To generate the parental genotypes, the egg collection cages were assembled as following:

• *w1118 x w1118*: The homozygote cross between *w1118* parents which generated the control parental genotype;
• *w1118 x uro*^*-*^ and *uro*^*-*^ *x w1118*: The heterozygote cross between w1118 and *uro*^*-*^ parents. I designed crosses which allowed for disentangling the effects of *uro*^*-*^ from mother or father (maternal or paternal effects). For the purpose of this study, which has a complex experimental design as it stands, I considered both paternal and maternal parental populations together as the heterozygote parental genotype w1118*/uro*^*-*^*.*
• *uro*^*-*^*x uro*^*-*^*:* The homozygote cross between *uro*^*-*^ parents which generated the *uro*^*-*^ parental genotype;

Egg collection cages were maintained in controlled environments as described above, and the oviposition device was changed every 8 to 12 h (with one exception in *Experiment 3* detailed below). Eggs obtained from the above crosses were then used in three experiments: *Experiment 1: Oviposition, Experiment 2: Development* and *Experiment 3: Mixed genotypes* (described below)*.* Note that these experiments were not run simultaneously because of logistic reasons as well as due to the sequential nature of the experimental design. Nonetheless, the methodology to obtain the parental genotypes were the same for all experiments.

#### Experiment 1: Oviposition

Ten *μ*L of eggs were collected for each parental genotype (see ‘Egg collection and parental crosses’ section) were deposited in vials containing 5 mL of the balanced diet, and allowed to develop until adult emergence. Upon emergence, 10 males and 10 females were transferred to fresh vials and allowed to mate for 4 consecutive days, during which the group was transferred to fresh vials every 2 days. In the fifth day, males were discarded, and 5 randomly selected females were selected, and placed in replicate vials containing 5 mL of one of the diet treatments described above (n = 6 per diet treatment per parental genotype, *n*_*total*_ *=* 216). Females were allowed to oviposit for 2 consecutive days, with fresh diets every day, before being discarded. Eggs were frozen in −20°C freezers and counted under a Leica M9 stereoscope as a proxy for female oviposition.

#### Experiment 2: Development

Eggs were collected for each parental genotype and squirted to vials containing 1 mL of each of the diet treatments at randomly generated volumes that were based on a standard curve generated during the preliminary stages of the experiment (see ‘Larval Crowding’ below; Figure S1). This approach allowed me to generate a (bimodal) continuous distribution of eggs deposited in each diet, which was informed by the natural history of the species as well as by the results found in the *Experiment 1*^45^ (see Figure S1). Egg volume was used as proxy for larval crowding as in other studies.^42^^,^^43^ Vials with eggs were allowed to develop to pupation, whereby the larval developmental time (in days) was scored from the day in which eggs were deposited until the day in which the first pupae appeared in the vial. Vials were discarded if first pupation did not occur within 30 days of seeding the eggs, and we scored these vials as ‘incomplete development’. Pupation success was scored as the total number of pupae that were formed in each vial, up to fifteen days after the first pupation. Pupation height was measured using a ruler that was attached to the table, upon which vials could be scanned and measured. I scored the height of all pupae attached to the wall of the vial. There were seven replicates per diet treatment per parental genotype (*n*_*total*_ *=* 252) (Figure 1). The majority of replicates in the 300 mM urea concentration died (average replicate deaths: 0 mM: 15.3%; 30 mM: 20.7%, 300 mM 72.2%). In fact, 100% of sugar-rich 300 mM larvae died. Thus, all of the statistical inferences were conducted excluding 300 mM urea treatment from the analysis (see also ‘STAR methods’).

#### Experiment 3: Mixed genotypes

To investigate whether or not uric acid modulated larval developmental speed in mixed genotype environments, I designed an assay inspired by previous studies.^82^^,^^83^^,^^84^ Ten *μ*L of eggs were collected within a time span of 2 h, from both the control w1118 (w1118 x w1118) and *uro*^*-*^ (*uro*^*-*^ *x uro*^*-*^) parental genotypes, and were jointly deposited in vials containing 5 mL of protein-rich, balanced, or sugar-rich diets with 0 mM (benign) and 300 mM (toxic) of urea (Figure 1) (*n*_*total*_ *=* 38). All larvae from the 300 mM died and, therefore, the results presented here pertain to the experiment on 0 mM only. Eggs were allowed to develop and vials were scanned every 4 h for adult emergence. Once the first adult emergence was recorded, vials were stored for an additional 4 h and then frozen in −20°C freezers. The number of adults of each genotype was scored, along with the total number of pupae in the vial. I then estimate the proportion of adults that emerged within the first 4 h from first adult emergence from each genotype as a proxy of speed in larval development. The assumptions of this assay are that (i) the competitive ability of the strains is unaffected by the phenotypic marker present in *uro* and that (ii) developmental time is a competitive trait with fitness implications. Assumption (ii) is valid as previous studies have extensively shown the role of developmental time in larval competition and fitness.^35^^,^^36^^,^^82^^,^^83^^,^^85^ However, assumption (i) might not be valid as previous studies show that *mini-white* influences *D. melanogaster* metabolism.^86^ The profile of responses of *w1118* and the heterozygotes *w1118/uro*^*-*^ are similar in this study, suggesting that there might not be a violation of assumption (i). Nonetheless, to circumvent this limitation, statements about developmental speed and the proportion of fast-emerging adults are made in comparative terms between w1118 and *uro*^*-*^ strains.

#### Larval crowding design

In Experiment 2, I developed a new protocol to generate larval crowding levels that are continuous (as opposed to the traditional ‘low’ vs ‘high’ categories). This was done as following: First, I collected the data from Experiment 1 on female oviposition behavior. Second, I used the natural history information on the larval crowding of a population of *D. melanogaster*.^45^ Based on the two values, I then generated a bimodal continuous distribution of eggs (proxy for larval crowding) where I deposited random volumes of egg-water solution determined by a simple computer algorithm and the standard curve for the relationship between the volume of egg-water solution and the number of eggs deposited (Figure S1A). The volumes were determined for each treatment (i.e., combination of diet x urea x genotype) separately. This methodology was applied for crosses and was successful generating different distributions of ‘crowding’ levels, whereby one of the median values matched median of the number of eggs laid in Experiment 1 and the other which matched the upper value of the larval crowding observed in a natural population; there was a difference between these two distributions generated by the method (*Crowding:* F_1, 432_ = 771.88, p < 0.001; Table S3). Thus, the bimodal distributions had median larval crowding levels that matched both the median oviposition from Experiment 1 (i.e., solid vertical line in Figure S1B) and the upper limit of the larval crowding observed in the natural population (i.e., 25 larvae or eggs/g or mL of diet)^45^ (dashed vertical line in Figure S1B). This is important because it demonstrate that the results observed here can be interpreted in light of the natural behavior of the flies and is ecologically significant. Practically, this approach enables continuous linear regressions to be fitted and therefore, increased the statistical power of the analysis.

### Quantification and statistical analysis

All analyses were conducted in R version 3.6.2.^87^ I used the ‘lme4’ and ‘lmerTest’ packages to fit random linear models.^88^^,^^89^ The majority of replicates in the 300 mM urea concentration died (average replicate deaths: 0 mM: 15.3%; 30 mM: 20.7%, 300 mM 72.2%). In some cases, 100% of sugar-rich 300 mM larvae died. To minimize biases that can be introduced due to very low sample sizes, I adopted a conservative approach and conducted all analysis excluding 300 mM urea concentration treatments from the statistical models. To analyze and compare uric acid concentration levels, I used a general linear regression with uric acid concentration (nmole per fly) as dependent variable and genotype as independent variable. In *Experiment 1,* I used LMM to control for the random effects of egg batch while investigating the fixed effects (and their interactions) of diet, urea concentration, and parental genotype. I also included linear and non-linear (quadratic) effects of urea concentration, and thus this variable had to be scaled to improve fit of the model. Likewise, the number of eggs laid was square-root-transformed to improve model fit. In *Experiment 2,* the models for developmental time, pupation height and pupation success controlled for the random effects of egg batch and the mixture of eggs that composed the population while investigating the fixed effects (and their interactions) of larval crowding, diet, urea concentration, and genotype. Developmental time was square-root-transformed, pupation height was transformed by elevating to the 0.25 power and egg number (as proxy for larval crowding) was natural-log-transformed (i.e., ln) to improve model fit. In *Experiment 3,* a GLM model was fitted with binomial family and *quasi* parameter to test for differences in the proportion of early emergence between diets, genotype, sex of the emerging adult, and their interactions. For all models, interactions were constructed *a priori* and were thus, kept in the final model even if not statistically significant. Data visualisation was done using the ‘ggplot2’ package.^90^

## Data Availability

Raw data have been deposited at Dryad and are publicly available as of the date of publication. DOIs are listed in the key resources table. Any additional information required to reanalyze the data reported in this paper is available from the lead contact upon request.

## Supplemental information

Document S1. Figure S1 and Tables S1–S4

Data S1. Oviposition, related to Figure 2

Data S2. Development, related to Figure 3

Data S3. Pupal height, related to Figure 3

Data S4. Mixed genotype, related to Figure 3
