# Supplementary material for: Uric acid metabolism modulates diet-dependent responses to intraspecific competition in Drosophila larvae
Source: iScience. 2022 Nov 15;25(12):105598. doi: 10.1016/j.isci.2022.105598 (PMC9706702; doi:10.1016/j.isci.2022.105598)
Supplement: Document S1. Figure S1 and Tables S1–S4 [file mmc1.pdf]

iScience, Volume *25*

Supplemental information

Uric acid metabolism modulates diet-dependent responses to intraspecific competition in *Drosophila* larvae

Juliano Morimoto

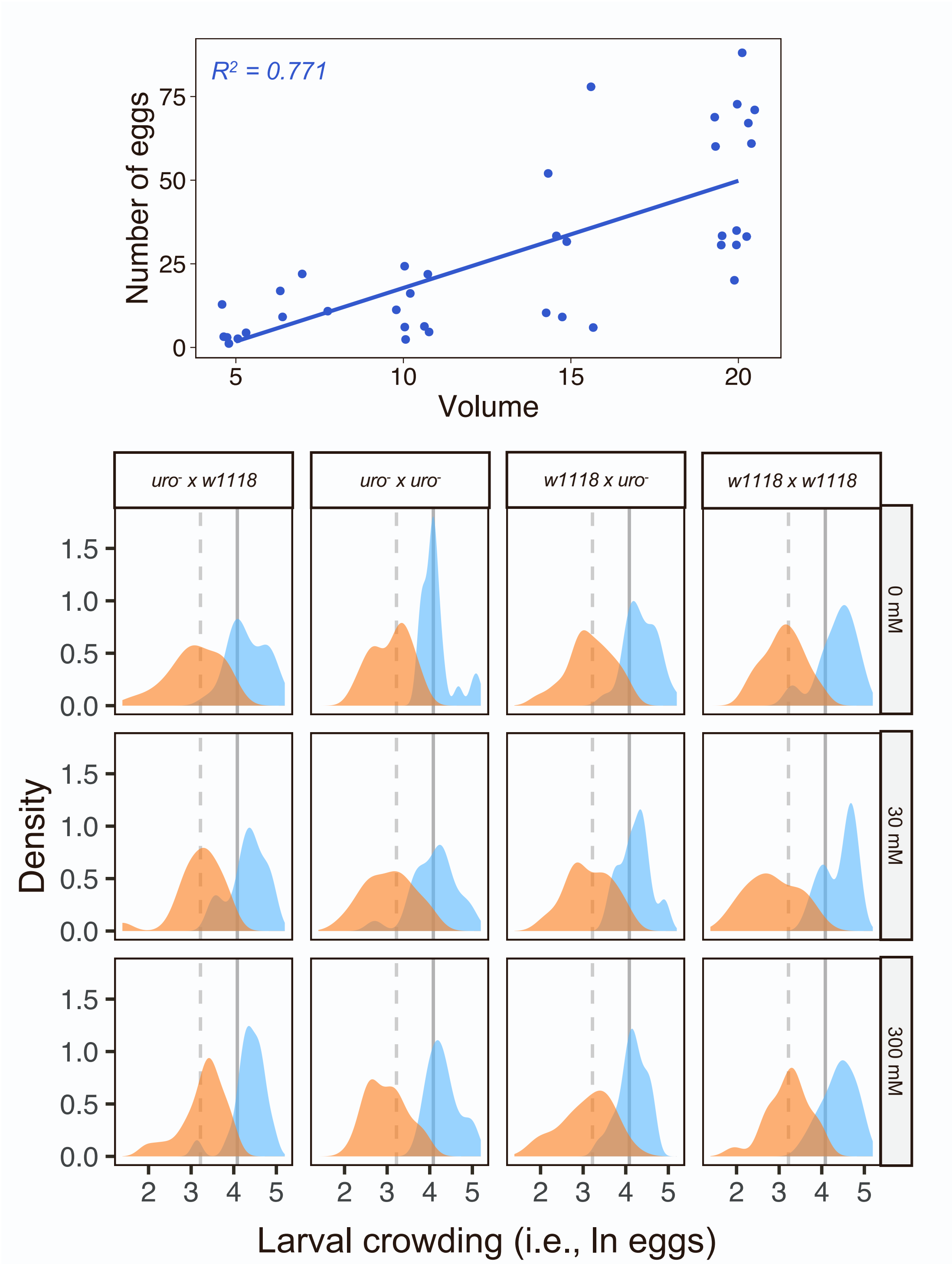

Figure S1. Manipulation of larval crowding (Related to STAR Methods and Figure 1). (Top panel) Standard curve for the relationship between the volume (in μL) of the egg-water solution and the number of eggs deposited in the substrate. (Bottom panel) The bimodal distributions created for larval crowding in this experiment. Dashed vertical line indicates the average larval crowding observed in nature [1] and vertical solid line indicates the average number of eggs laid in *Experiment 1* (see also). Note the similarity of the distributions, as well as their coverage as a continuous variable.

Table S1. Diet recipes (Related to STAR Methods).

Ingredients	Balanced	Sugar-rich	Protein-rich
Agar	0.73	0.73	0.73
Brewers Yeast	2.5	1.09	5.41
Sucrose	4	5.41	1.09
Nipagin (stock 25x)	0.5	0.5	0.5
Urea	0/0.09/0.18	0/0.09/0.18	0/0.09/0.18
Water (complete)	50	50	50

Table S2. Female oviposition analysis (Related to Figure 2). Bold $p < 0.05$. See also Figure 2.

	Sum Sq	Mean Sq	NumDF	DenDF	F-value	p-value
**Diet**	6165.3	3082.6	2	199	8.6673	**<0.001**
**[Urea]**	292.3	292.3	1	199	0.822	0.3657
**[Urea]^2**	3485.8	3485.8	1	199	9.8009	**<0.001**
**Genotype**	1075.7	358.6	3	199	1.0081	0.3902
**Diet * [Urea]^2**	2532.2	1266.1	2	199	3.5598	**<0.001**
**[Urea]^2*Genotype**	1084.9	361.6	3	199	1.0168	0.3863
**Diet * [Urea]**	724.7	362.3	2	199	1.0187	0.3629

Table S3. Analysis of the methodology (Related to STAR Methods and Figure 1). Bold $p < 0.05$.

	Df	Sum Sq	Mean Sq	F-value	p-value
**Diet**	2	0.685	0.343	1.4856	0.2275
**[Urea]**	2	0.382	0.191	0.8294	0.437
**Genotype**	3	1.488	0.496	2.152	0.0931
**Crowding (L vs H)**	1	177.965	177.97	771.88	**<0.001**
**Diet*[Urea]**	4	1.786	0.446	1.9365	0.1034
**Diet*Genotype**	6	2.072	0.345	1.498	0.1772
**Genotype*[Urea]**	6	1.119	0.186	0.8086	0.5636
**Diet:Crowding**	2	0.267	0.133	0.578	0.5615
**Crowding*[Urea]**	2	0.042	0.021	0.0906	0.9134
**Genotype*Crowding**	3	0.902	0.301	1.3043	0.2725
**Diet*[Urea]*Genotype**	12	0.637	0.053	0.2304	0.9969
**Diet*Crowding*[Urea]**	4	0.187	0.047	0.2025	0.937
**Diet*Genotype*Crowding**	6	2.845	0.474	2.0563	0.0572
**Crowding*[Urea]*Genotype**	6	1.567	0.261	1.1325	0.3423
**Diet*Crowding*[Urea]*Genotype**	12	0.939	0.078	0.3394	0.9816
**Residuals**	432	99.602	0.231		

Table S4. Complete life-history statistics (Related to STAR Methods and Figure 3).

Trait	Factors	Sum Sq	Mean Sq	NumDF	DenDF	F-value	p-value
**Developmental time**	**Genotype**	0.071	0.035	2	270	1.443	0.238
	**[Urea]**	0	0	1	269.6	0.001	0.973
	**log(eggs) (Density)**	0.658	0.658	1	268.1	26.792	**<0.001**
	**Diet**	160.6	80.302	2	270.3	3269.84	**<0.001**
	**Genotype:[Urea]**	0.05	0.025	2	269.6	1.015	0.364
	**Genotype:log(eggs)**	0.097	0.048	2	270.6	1.973	0.141
	**[Urea]:log(eggs)**	0.075	0.075	1	271	3.044	0.082
	**Genotype:Diet**	0.321	0.08	4	269.7	3.263	**0.012**
	**[Urea]:Diet**	0.353	0.176	2	269.4	7.179	**<0.001**
	**log(eggs):Diet**	0.023	0.011	2	269.7	0.466	0.628
	**Genotype:[Urea]:log(eggs)**	0.005	0.002	2	270.8	0.097	0.908
	**Genotype:[Urea]:Diet**	0.038	0.009	4	269.5	0.384	0.820
	**Genotype:log(eggs):Diet**	0.5	0.125	4	270	5.086	**<0.001**
	**[Urea]:log(eggs):Diet**	0.016	0.008	2	270	0.321	0.726
	**Genotype:[Urea]:log(eggs):Diet**	0.128	0.032	4	270	1.303	0.269
**Pupation height**	**Genotype**	0.0075	0.0038	2	243.4	0.201	0.818
	**[Urea]**	0.0257	0.0257	1	243.1	1.380	0.241
	**log(eggs) (Density)**	1.0937	1.0937	1	244.8	58.715	**<0.001**
	**Diet**	0.0075	0.0037	2	243.8	0.201	0.818
	**Genotype:[Urea]**	0.0532	0.0266	2	243.2	1.427	0.242
	**Genotype:log(eggs)**	0.1415	0.0707	2	243.4	3.798	**0.024**
	**[Urea]:log(eggs)**	0.002	0.002	1	243.4	0.106	0.745
	**Genotype:Diet**	0.0507	0.0127	4	243.2	0.681	0.606
	**[Urea]:Diet**	0.1402	0.0701	2	243.1	3.764	**0.025**
	**log(eggs):Diet**	0.0091	0.0046	2	243.3	0.245	0.783
	**Genotype:[Urea]:log(eggs)**	0.0675	0.0337	2	243.2	1.812	0.166
	**Genotype:[Urea]:Diet**	0.0778	0.0194	4	243.1	1.044	0.385
	**Genotype:log(eggs):Diet**	0.1798	0.0449	4	243.4	2.413	**0.049**
	**[Urea]:log(eggs):Diet**	0.0329	0.0164	2	243.3	0.883	0.415
	**Genotype:[Urea]:log(eggs):Diet**	0.056	0.014	4	243.2	0.752	0.558
**Pupation success**	**Genotype**	49.3	24.6	2	269.1	1.137	0.322
	**[Urea]**	0.1	0.1	1	269.1	0.004	0.949
	**log(eggs) (Density)**	4488	4488	1	270	207.181	**<0.001**
	**Diet**	579.6	289.8	2	269.4	13.379	**<0.001**
	**Genotype:[Urea]**	9	4.5	2	269.1	0.209	0.812
	**Genotype:log(eggs)**	221.1	110.6	2	269.3	5.105	**0.007**
	**[Urea]:log(eggs)**	1.2	1.2	1	269.3	0.057	0.812
	**Genotype:Diet**	186.9	46.7	4	269.1	2.157	0.074
	**[Urea]:Diet**	100.9	50.5	2	269.1	2.329	0.099
	**log(eggs):Diet**	35.4	17.7	2	269.2	0.818	0.442
	**Genotype:[Urea]:log(eggs)**	52.9	26.4	2	269.3	1.221	0.297
	**Genotype:[Urea]:Diet**	48.2	12	4	269.1	0.556	0.695
	**Genotype:log(eggs):Diet**	183.7	45.9	4	269.2	2.120	0.079
	**[Urea]:log(eggs):Diet**	38.6	19.3	2	269.2	0.892	0.411
	**Genotype:[Urea]:log(eggs):Diet**	63.3	15.8	4	269.2	0.731	0.572
